# Hydrothermal liquefaction aqueous phase mycoremediation to increase inorganic nitrogen availability

**DOI:** 10.1016/j.heliyon.2024.e31992

**Published:** 2024-05-27

**Authors:** Vitoria F.C. Leme, Karla Lopez, Tiago Costa, Beth Conerty, Laurie B. Leonelli, Yuanhui Zhang, Paul C. Davidson

**Affiliations:** Agricultural & Biological Engineering, University of Illinois Urbana-Champaign, Urbana, IL, 61801, USA

**Keywords:** Hydrothermal liquefaction aqueous phase, *Trametes versicolor*, Mycoremediation, Nutrient recovery, Bioremediation

## Abstract

Hydrothermal liquefaction aqueous phase (HTL-AP) is a waste product from a thermochemical process where wet biomass is converted into biocrude oil. This nutrient-rich wastewater may be repurposed to benefit society by assisting crop growth after adequate treatment to increase inorganic nitrogen, especially NO_3_^−^. This study aims to increase HTL-AP inorganic nitrogen, specifically NH_3_/NH_4_^+^ and NO_3_^−^, through fungal remediation for further use in hydroponic systems. *Trametes versicolor*, a white-rot fungus known for degrading a range of organic pollutants, was used to treat a diluted (5 %) HTL-AP for 9 days. No fungal growth was observed, but *T. versicolor* activity was suspected by laccase activity throughout cultivation time. NO_3_^−^-N and NH_3_/NH_4_^+^-N increased by 17 and 8 times after three days of fungal treatment, which was chosen as the appropriate time for HTL-AP fungal treatment as it resulted in the highest concentration of NO_3_^−^-N. The addition of nitrifying bacteria to the fungal treatment resulted in a twofold increase in NO_3_^−^-N concentration compared to the fungal treatment alone, indicating an enhancement in treatment efficacy. COD decreased by 51.33 % after 24 h, which may be related to the fungus’ capacity to reduce the concentration of organics in the wastewater; nonetheless, COD increased in the following days, which may be related to the release of fungal byproducts. *T. versicolor* shows promise as a potential candidate for increasing inorganic nitrogen in HTL-AP. However, future studies should primarily address HTL-AP toxicity, reducing NH_3_/NH_4_^+^-N while increasing NO_3_^−^-N, and hydroponics crop production after fungal treatment.

## Highlights

• Fungal treatment increased HTL-AP NO_3_^−^-N and NH_3_/NH_4_^+^-N concentrations by 17- and 8-times after three days of treatment.
• An increase in laccase activity was observed during fungal cultivation in HTL-AP.
• The NO_3_^−^-N concentration peaked after three days of fungal treatment.
• The addition of nitrifying bacteria improved the HTL-AP fungal treatment by doubling NO_3_^−^-N concentration.

## 1 Introduction

Fossil fuels' extensive use has introduced environmental problems around the world, directing attention to alternative fuels [1,2]. For instance, the hydrothermal liquefaction (HTL) of biomass has drawn attention as an alternative method for producing biocrude oil, which can be further upgraded to transportation biofuel [1,3]. This process can use a range of biomasses as feedstock, including waste products such as food waste [4]. The Office of Resource Conservation and Recovery [5] estimated the generation of around 103 million tons of food waste in the United States in 2018. Hence, HTL is a potential solution to deal with two issues modern society faces: fossil fuels and food waste generation. However, a complex and potentially toxic liquid stream called hydrothermal liquefaction aqueous phase (HTL-AP) is generated as a byproduct that needs to be treated before discharged into the environment [6]. HTL-AP may contain heavy metals and has a high concentration of organics, including nitrogen-containing organics and aromatic compounds, that impose challenges on its treatment or further use [4]. Alternatively, HTL-AP has the potential for reuse in hydroponic systems after adequate treatment, as it is free from pathogens due to the high temperature and pressure conditions of the HTL process, yet contains nutrients that can support crop growth [7]. Nonetheless, most of the nitrogen in HTL-AP from swine manure HTL is in organic form and not readily accessible to plants, which uptake nitrogen as ions (e.g., NH_4_^+^ and NO_3_^−^) [8,9]. This underscores the need for new studies that seek to increase inorganic nitrogen availability in HTL-AP to increase the overall value of the HTL process.

Biological treatment of HTL-AP has been explored and possesses several advantages, such as its eco-friendly nature, cost-effectiveness, lower energy consumption compared to traditional methods, and ability to reduce toxicity, coupled with the potential to produce value-added compounds [4,10]. He et al. [11] explored different bacteria strains from *Rhodococci* sp. to produce lipid-rich bacterial biomass from algae or woody substrates when grown in HTL-AP. Si et al. [12] sought to produce biohythane from HTL-AP using two-stage fermentation, where hydrogen gas and organic acids were produced in the first step by substrate hydrolyzation, and the organic acids transformation further generated methane. Chen et al. [13] assessed algae biomass production from HTL-AP. Similarly, Yang et al. [14] tested algae biomass cultivation with HTL-AP that was pre-treated using anaerobic digestion as a detoxification step and for methane production. Cordova et al. [15] used engineered yeast strains to produce the value-added chemicals itaconic acid and triacetic acid lactone using HTL-AP as a complementary nutrient source. Bioelectrochemical systems were also explored for HTL-AP treatment for electricity and hydrogen generation [16,17]. All the mentioned studies indicate that microorganisms can grow using HTL-AP as a nutrient source and produce value-added compounds. However, none of them focused on increasing inorganic nitrogen in this wastewater.

Emerging as an alternative biological treatment, fungal treatment of HTL-AP may increase inorganic nitrogen availability while also reducing its toxicity, which is mainly related to the presence of heavy metals and organic nitrogen molecules, aromatic or not [4,18]. Fungal treatment, also called mycoremediation, is an eco-friendly approach where fungal species degrade, remove, or convert contaminants from wastewater through different mechanisms [19]. Fungi can uptake small organic nitrogen compounds and release their excess as NH_3_/NH_4_^+^ through ammonification [20,21]. Additionally, fungal mycelium has shown a significant sorption capacity to remove heavy metals and organic molecules from liquid streams, and some fungal species, specifically from the white-rot fungus group, can excrete enzymes (e.g., laccase) that have the capacity to degrade a range of organic nitrogen pollutants [[22], [23], [24], [25], [26]]. These microorganisms tend to excrete these enzymes to transform complex nutrients into simpler ones, making them easier to utilize [27]. Therefore, mycoremediation may be a potential alternative to increase inorganic nitrogen while reducing HTL-AP toxicity, aiming for future use of this waste stream as fertilizer in hydroponic systems.

Among all fungal species, white-rot fungi have stood out as a promising fungal group for pollutant removal due to their enzymatic machinery and stability under adverse conditions [28,29]. They have been explored for the mycoremediation of a range of recalcitrant organic pollutants, such as dyes, pharmaceutical compounds, phenols, and pesticides [28]. *Trametes versicolor* is the most widely investigated fungus from this group and has been reported to treat wastewater containing complex organic pollutants and heavy metals [23,30,31]. While *T. versicolor* has been explored for other wastewaters and in combination with other biological treatments [32], there are no studies addressing how long the fungus should be cultivated in HTL-AP. In this context, to the best of the authors’ knowledge, this is the first study addressing *T. versicolor* optimal cultivation time when used as the sole source of treatment to increase inorganic nitrogen in HTL-AP. Therefore, the contribution of this study is to investigate the increase of inorganic nitrogen in HTL-AP by cultivating the fungus *T. versicolor* in this wastewater, thereby facilitating the potential utilization of this waste stream as a fertilizer in hydroponic systems. Several parameters (NH_3_/NH_4_^+^-nitrogen, NO_3_^−^-nitrogen, COD, biomass dry weight, and laccase activity) were evaluated across 9 days of fungal cultivation to determine the effect of mycoremediation on HTL-AP for increasing inorganic nitrogen concentration, as well as the time needed for maximum treatment effectiveness. Additionally, fungal treatment along with nitrifying bacteria was explored to evaluate the increase in NO_3_^−^ concentration.

## 2 Material and methods

### 2.1 Hydrothermal liquefaction aqueous phase (HTL-AP)

HTL-AP was provided by the Environment-Enhancing Energy (E2-E) Laboratory (University of Illinois at Urbana-Champaign) from the hydrothermal liquefaction of food waste (at 280 °C, 30 min retention time). The raw HTL-AP was stored at 4 °C until used and its biochemical properties are described in Table 1. For these experiments, HTL-AP was diluted to 5 % in DI water and stored at 4 °C. The 5 % dilution was chosen based on a previous study conducted by Jesse et al. [8], which evaluated hydroponic lettuce production using 2.5 % HTL-AP from swine manure. While some crop growth was observed when using the 2.5 % HTL-AP, the growth was less than the crops receiving a standard hydroponic fertilizer. Therefore, the concentration in this study was doubled to utilize a higher percentage of HTL-AP but still low enough for future evaluation in hydroponic leafy green crop production.

**Table 1:** HTL-AP characteristics. NH_3_/NH_4_^+^-N: ammonia/ammonium-nitrogen, NO_3_^−^-N: nitrate-nitrogen, and COD: chemical oxygen demand.

HTL-AP	NH_3_/NH_4_^+^-N (mg/L)	pH	NO_3_^−^-N (mg/L)	COD (mg/L)
Raw	80.86 ± 4.02	4.09 ± 0.01	36.80 ± 1.70	9909.33 ± 52.09
5 %	4.04 ± 0.20	4.29 ± 0.51	1.84 ± 0.08	495.47 ± 2.60

### 2.2 Fungal strain

*Trametes versicolor* was kindly donated by the Miller Mycology Lab – Illinois Natural History Survey (University of Illinois at Urbana-Champaign). The fungal species was routinely cultivated in sterile potato dextrose agar (PDA) medium (15 psi, 121 °C, 15 min) at 28 °C for 5–7 days and stored at 4 °C until use.

### 2.3 Mycelial suspension

The mycelial suspension was prepared according to Blánquez et al. [33] with some modifications. Briefly, a 500 mL Erlenmeyer containing 150 mL sterile malt extract medium (ME, 2 % w/v, 15 psi, 121 °C, 15 min) was inoculated with 4 mycelial plugs (1 cm diameter) from the fungus growing region on PDA. The flask was sealed with a gauze filter followed by a KC100 sterilization wrap and incubated for 4 days at 22 ± 1 °C and 135 rpm. The resulting thick mycelial plugs were washed twice with sterile DI water (15 psi, 121 °C, 15 min), and immersed in sterile 0.85 % NaCl (15 psi, 121 °C, 15 min). Lastly, the fungal plugs in saline solution were ground and mixed using a bead beater homogenizer by the addition of 6 matrix M beads (MP biomedicals; Irvin, California, U.S.A.; 60 s at 6.5 m/s). The mycelial suspension was stored at 4 °C until use.

### 2.4 Pellets production

Fungal pellets were produced following modified methods from Blánquez et al. [33]. Briefly, 1 mL of mycelial suspension was added to a 1 L flask containing 250 mL of sterile malt extract medium (2 % w/v). The samples were incubated at 135 rpm and 22 ± 1 °C for 6 days. The pellets were transferred aseptically to centrifuge tubes (50 mL), washed twice with sterile DI water, and stored at 4 °C with the addition of sterile saline solution (0.85 % NaCl) until use.

### 2.5 Wastewater time-experiment

The wastewater time-experiment was carried out in triplicates in 250 mL flasks containing 50 mL of 5 % sterile HTL-AP (v/v, 121 °C, 15 min) inoculated with 6.4 % (w/v) wet fungal pellets. The HTL-AP concentration was selected based on findings from prior research. Jesse et al. (2019) successfully demonstrated lettuce growth in treated 2.5 % HTL-AP [8]. Here, 5 % is proposed to increase the HTL-AP concentration that can be used to grow crops successfully. Positive controls (duplicates) were prepared with 2 % (w/v) malt extract broth and negative controls (triplicates) with 5 % HTL-AP without inoculation. Negative controls were used for comparison to the treated samples. The samples were then incubated (135 rpm, 28 °C) and collected according to the time scheduled (4, 24, 46, 72, 96, 118, 168, and 214 h). While negative controls were obtained for each time data point, positive controls were analyzed after 214 h of cultivation time. After the incubation period, the samples were filtered (Whatman 42) and stored at 4 °C until analyzed. For dry biomass, fungal pellets were dried at 70 °C for 2 h and 10 min. Lastly, HTL-AP pH was recorded before and after fungal cultivation.

### 2.6 Wastewater nitrifying experiment

The wastewater nitrifying experiments were carried out in triplicates in 250 mL flasks containing 50 mL of 5 % sterile HTL-AP (v/v, 121 °C, 15 min). Flasks were aseptically inoculated with either 6.4 % (w/v) wet fungal pellets (Tv-5HTL-AP) or 190 μL ATM Aquarium Products Colony Nitrifying Bacteria (ATM Aquarium Products, Las Vegas, NV, USA) containing the bacteria *Nitrosomonas and Nitrobacter* (B-5HTL-AP), or the combination of both (B + Tv-5HTL-AP). Negative controls were prepared and labeled as 5HTL-AP. The samples were incubated for 3 days at 28 °C and 135 rpm. Following incubation, the samples were filtered (0.45 μm) and stored at 4 °C until analyzed.

### 2.7 Wastewater characterization

#### 2.7.1 Nutrients analysis

Measurements for ammonia/ammonium-nitrogen (NH_3_/NH_4_^+^-N), nitrate-nitrogen (NO_3_^−^-N), and chemical oxygen demand (COD) were performed according to Hach methodologies 8038, 8039, and 8000, respectively. For each measurement, at least triplicates were analyzed. Additionally, for NO_3_^−^-N and COD measurements, only two negative controls were randomly selected for analysis. NO_3_^−^-nitrogen and NH_3_/NH_4_^+^-nitrogen readings were performed using Hach DR/2010 spectrophotometer (Loveland, Colorado, U.S.A.), while Hach DR/3900 (Loveland, Colorado, U.S.A.) was used for COD. The results are presented as the average of the readings with their respective standard deviations.

#### 2.7.2 Enzyme assay

Laccase activity was determined as previously described by Majcherczyk et al. [34] and Boujelben et al. [30]. Briefly, samples were added to a solution containing 1 mM 2,2-azino-bis- [3-ethylthiazoline-6-sulfonate] (ABTS) in 0.1 M Na-tartrate buffer (pH 4.5) and monitored at 420 nm of absorbance in a 1 cm pathlength cuvette. The rate at which 1 μmol of ABTS is oxidized per minute was used as the unit of laccase activity (1 U = 1 μmol ABTS oxidized min^−1^; ε_420_ = 36,000 M^−1^ cm^−1^) [34].

#### 2.7.3 Statistical analysis

An ANOVA was utilized to investigate the effects of time of incubation and fungal treatment on COD and NO_3_^−^-N. The objective was to determine if there were significant differences in mean levels of COD and NO_3_^−^-N across various time points and fungal treatments. Furthermore, a Kruskal-Wallis test was employed to evaluate the effects of time of incubation and fungal treatment on NH_3_/NH_4_^+^-N. This non-parametric test was preferred as the distribution of NH_3_/NH_4_^+^-N levels did not meet the normality assumptions needed for ANOVA.

## 3 Results and discussion

### 3.1 Wastewater time-experiment

**Fig. 1:**
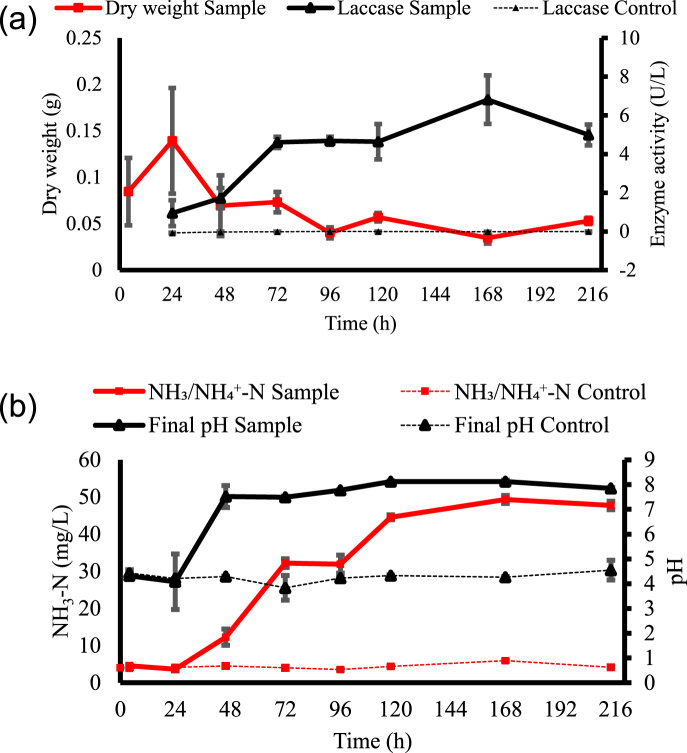
Responses of *T. versicolor* treatment compared to the negative control: (a) Fungal dry weight and laccase activity and (b) NH_3_/NH_4_^+^-N and pH measured during *T. versicolor* cultivation in 5 % HTL-AP over time.

Changes in pH, NH_3_/NH_4_^+^-N, NO_3_^−^-N, COD, dry weight, and laccase activity were monitored over time to assess the impact of fungal cultivation on wastewater treatment. Results for dry weight and laccase activity are presented in Fig. 1 (a). Fungal dry biomass slightly decreased over time (Fig. 1, a). Cruz-Morató et al. [35] reported a similar behavior and explained it as the lack of nutrients in the medium, which may cause mycelium lysis and a further decrease in biomass. While a low dry mass was observed in HTL-AP inoculated samples (<0.14 g) during the cultivation period, the positive control dry mass after 214 h resulted in 2.05 ± 0.03 g of dry biomass, implying the activity of the fungal inoculum employed in this experiment. Enzyme activity by *T. versicolor* increased over time (Fig. 1, a), suggesting fungal activity during wastewater cultivation [30]. No activity was detected in the negative controls, suggesting that laccase activity was solely attributed to the presence of *T. versicolor,* as all samples were autoclaved prior to the start of the experiment, and hence, no other biological activity was present. Similar behavior was described by Blánquez et al. [36], who claimed that *T. versicolor*, under nutrient limitation, can produce laccase while no growth is observed. In fact, no growth may benefit HTL-AP fungal treatment when the goal is to increase the fertilizer value, rather than increase fungal biomass. For instance, excessive fungal biomass on the reactor's walls would be avoided, which is known to be a problem in larger bioreactors [36]. In addition, the laccase enzyme released by the fungus may assist in removing potential toxic compounds present in the wastewater as it is known to degrade complex organics [28]. Thus, the absence of increased *T. versicolor* biomass when cultivated in HTL-AP may be advantageous to this wastewater treatment for further use in hydroponic systems, and laccase production may reduce the wastewater toxicity.

Hydroponically grown plants uptake nitrogen as ions, i.e., NH_4_^+^ and NO_3_^−^ [9]. In these terms, measurements were taken to monitor any changes in these nitrogen ions during fungal cultivation. These data are important for two reasons: to evaluate whether *T. versicolor* can increase inorganic nitrogen in HTL-AP, and how long fungal cultivation should be performed to maximize concentrations of these nitrogen forms. There is a gradual increase in NH_3_/NH_4_^+^-N (Fig. 1, b). No changes in the negative controls for NH_3_/NH_4_^+^-N concentrations were observed, indicating that the increase in NH_3_/NH_4_^+^ concentration were solely attributed to the presence of *T. versicolor*. The time points of 168 and 214 h presented the highest values measured. They are significantly higher than the others but not significantly different from each other, according to the Kruskal-Wallis test (Table 2). After two days of fungal cultivation, NH_3_/NH_4_^+^-N increased from 4.04 mg/L to 12.30 mg/L, which aligns with the increase in pH during the same period (Fig. 1, b). HTL-AP pH after two days of fungal cultivation reached 7.52 and remained high during the rest of the days. Depending on the substrate C:N ratio, fungi can metabolize low molecular weight nitrogen organics and release NH_3_/NH_4_^+^ in this process, known as ammonification [20,21]. It is likely that *T. versicolor* performed ammonification, metabolizing short nitrogen organic chains and releasing the excess of nitrogen in the wastewater as NH_3_/NH_4_^+^; this might explain the reason for the NH_3_/NH_4_^+^-N increase over time. It is also possible that *T. versicolor* may prefer other sources of nitrogen when cultivated in wastewater rather than NH_3_/NH_4_^+^ nitrogen. As a result, NH_4_^+^ produced during ammonification would not be used by the fungus, which may explain the increase in NH_3_/NH_4_^+^-N in the samples (Fig. 1, b). NH_3_/NH_4_^+^-N measured during the experiments accounts for both NH_3_ and its ionic form (NH_4_^+^); NH_3_/NH_4_^+^ are in equilibrium, and their concentration depends on the solution pH [37]. Considering the pH of HTL-AP after fungal cultivation, and room temperature, it is possible to say that most of the NH_3_/NH_4_^+^-N in the wastewater after treatment is in its ionic (NH_4_^+^) form, which is preferred by hydroponically grown plants, since pKa is higher than pH (pKa = 9.25, pH = 7.52–8.12 [38]). An increase in NH_4_^+^ during *T. versicolor* wastewater treatment has been reported by Dalecka et al. [39] when treating non-sterile municipal wastewater. They observed an increase in NH_4_^+^ when wastewater pH was not adjusted (pH = 7.5–7.6), and no NH_4_^+^ changes occurred when wastewater pH was adjusted to pH = 5.5. Similarly, Hultberg and Bodin [40] stated that no changes happened in NH_4_^+^ concentration when treating brewery wastewater with pH = 6.6.

**Table 2:** NO_3_^−^-N, NH_3_/NH_4_^+^-N, and COD concentrations along fungal treatment time with their respective statistical analysis (Tukey HSD or Kruskal-Wallis test).

Time (h)	NO_3_^−^-N		NH_3_/NH_4_^+^-N		COD	
	mg/L	Tukey HSD	mg/L	Kruskal-Wallis	mg/L	Tukey HSD
24	6.1 ± 2.5	d	3.7 ± 0.5	f	241.1 ± 51.9	a
46	10.4 ± 2.5	cd	12.3 ± 2.2	d	255.2 ± 22.9	a
72	30.7 ± 2.1	a	32.2 ± 1.2	c	418.6 ± 8.1	b
94	29.3 ± 1.5	a	31.9 ± 2.4	c	417.8 ± 8.7	b
118	19.9 ± 3.5	b	44.5 ± 0.7	b	430.6 ± 19.2	b
168	17.8 ± 1.1	bc	49.3 ± 1.1	a	455.7 ± 32.1	b
214	20 ± 1.1	b	47.7 ± 1.1	a	525.7 ± 8.3	b

Note: within each column, means that are followed by the same letter are not significantly different (*p* < 0.05).

**Fig. 2:**
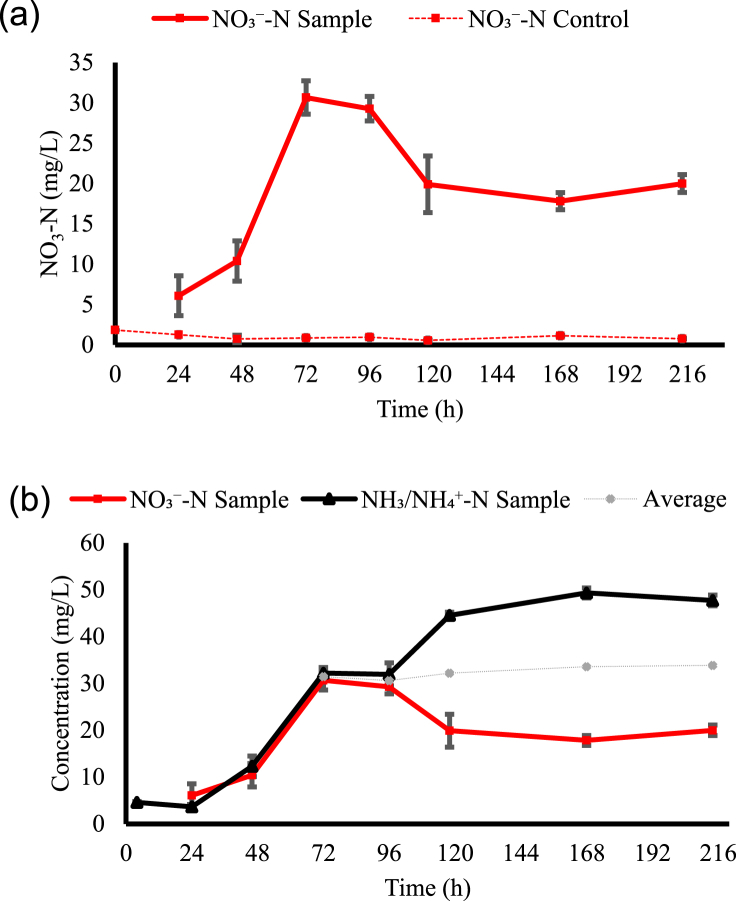
(a) NO_3_^−^-N and (b) NO_3_^−^-N and NH_3_/NH_4_^+^-N measured during *T. versicolor* cultivation in 5 % HTL-AP over time; average represents the average between NO_3_^−^-N and NH_3_/NH_4_^+^-N concentration at the same time; NO_3_–N control refers to the negative control.

NO_3_^−^-N results are found in Fig. 2 (a). An increase in NO_3_^−^-N is observed over the first 3 days, peaking at 30.67 mg/L. This shows a remarkable increase of 17 times in NO_3_^−^ concentration compared to the initial concentration in 5 % HTL-AP. No changes in the negative controls for NO_3_^−^-N concentrations were observed, implying that the increase in NO_3_^−^ concentration was solely due to the presence of *T. versicolor*. The time points of 72 and 94 h had a significantly higher NO_3_^−^-N than all the others, but they were not statistically significant from each other (Table 2). Nitrification has been reported in different filamentous fungi [41]. However, since no decrease in NH_3_/NH_4_^+^-N was observed during the cultivation period, the formation of NO_3_^−^-N from NH_4_^+^-N was not likely. Oxidation of NH_3_/NH_4_^+^-N or nitrogen-organics by these microbes, which includes recalcitrant compounds and small chains, may result in NO_3_^−^ generation; however, not much information exists about specific mechanisms responsible for this in fungi [42]. In these terms, it is speculated that *T. versicolor* may have performed nitrification using nitrogen organics over the first three days of cultivation. On the fourth day of fungal treatment, a decline in NO_3_^−^-N concentration is observed. Fig. 2 (b) shows that this decline occurred simultaneously with the rise in NH_3_/NH_4_^+^-N. Interestingly, adding the average between NO_3_^−^-N and NH_3_/NH_4_^+^-N concentrations at each specific time to Fig. 2 (b), it is noticed that after the third day, the increase in NH_3_/NH_4_^+^-N concentration seems to reflect the decrease in NO_3_^−^-N. Some fungi may uptake NO_3_^−^ as a nitrogen source for growth and release NH_4_^+^ [27,43]. However, specific enzymes, i.e., nitrate reductases, are necessary for fungal NO_3_^−^ uptake and further NH_4_^+^ generation [44]; NO_3_^−^-N assimilation by fungi may only take place in the absence of other preferred nitrogen sources [45]. Although it seems *T. versicolor* did not prefer NH_3_/NH_4_^+^-N as its primary nitrogen source, it is not possible to confirm whether this fungus produced the required enzymes to assimilate NO_3_^−^. In addition, as no growth could be observed (Fig. 2), there is no evidence that NO_3_^−^-N assimilation was used for growth. Still, it is hypothesized that the fungus consumed NO_3_^−^ in the absence of another preferred nitrogen source.

The temporal study aimed to evaluate important parameters during HTL-AP fungal treatment; the *T. versicolor* cultivation period was one of them. Hydroponic plants can absorb both NH_4_^+^ and NO_3_^−^ as nutrients, but excess of the former is known to harm them [9,46]. In this study, NO_3_^−^-N peaks on the third day of fungal cultivation (30.67 mg/L); on the same day, the pH reached 7.49 and the NH_3_/NH_4_^+^-N concentration was 30.20 mg/L, 8 times higher than the starting concentration. In addition, an increase in NO_3_^−^ concentration may benefit the future use of the treated wastewater as a fertilizer source. The NO_3_^−^/NH_4_^+^ ratio has been reported to impact hydroponic systems; an increase in the ratio tends to improve hydroponic plant growth [47,48]. Nitrifying bacteria could be used to increase the NO_3_^−^/NH_4_^+^ ratio of the treated HTL-AP. Nitrifying bacteria from *Nitrosomonas* and *Nitrobacter* genus can assist NH_4_^+^ conversion into NO_3_^−^ [9,49]. Bacterial nitrification was used by Jesse and Davidson [7] to increase NO_3_^−^ and nitrite (NO_2_^−^) in 5 % HTL-AP. An increase of only 1.75 mg/L of NO_3_^−^ + NO_2_^−^ was achieved, but HTL-AP toxicity or insufficient nitrification time may have directly impacted this small increase. Here, fungal treatment for three days increased the NO_3_^−^-N concentration by 28.83 mg/L. In addition, fungi possess the ability to remove pollutants, such as aromatic/nitrogen organics and heavy metals, that are known to be responsible for HTL-AP toxicity [4,28], which may improve nitrifying bacteria performance after *T. versicolor* treatment. Finally, three days of HTL-AP fungal treatment appears to provide the highest NO_3_^−^-N concentration; subsequently adding nitrifying bacteria could then further increase the NO_3_^−^ concentration by converting NH_4_^+^ to NO_3_^−^.

**Fig. 3:**
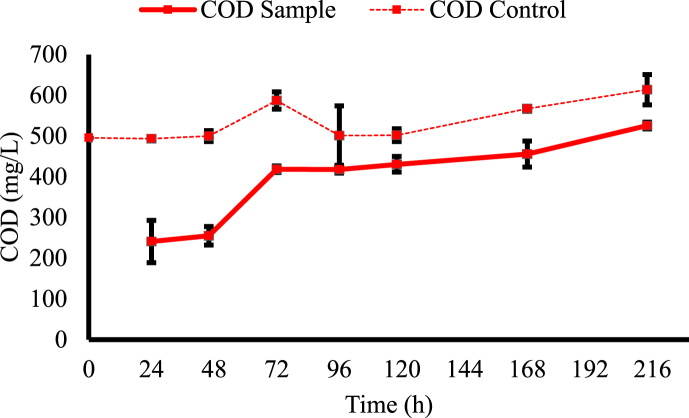
COD measured during *T. versicolor* cultivation in 5 % HTL-AP along the time compared to the negative control.

The COD analysis relates to the number of organics and the level of pollution in the liquid [50]. The COD analysis is shown in Fig. 3 and Table 2. In the first 24 h, *T. versicolor* removed 51.33 % of COD. This reduction was expected and has been reported by other studies. Cerrone et al. [51] demonstrated a 72 % COD removal when using this fungus to treat olive-washing wastewater. Similarly, Hultberg and Bodin [40] achieved a 67.1 % COD removal when treating brewery wastewater for 13 days. Boujelben et al. [30] reported COD removal from tannery wastewater by *T. versicolor* alive (31.2–45 %) and dead (19 %), showing the fungus’ capacity to adsorb organic pollutants into its mycelium structure. Nonetheless, after two days of treatment, COD increased and remained constant for several days. The time points from 72 to 214 h presented significantly higher COD values than of times points 24 and 46 h (Table 2). Boujelben et al. [30] reported an increase in COD after 7 days of fungal cultivation. They claimed this increase might be due to a lack of nutrients and the production of enzymes or metabolites by the fungus. In addition, constant COD has also been reported in the literature when treating urban wastewater by Cruz-Morató et al. [35]. It should be noted that the rise in COD may affect plant growth and could be linked to the formation of byproducts, such as enzymes and metabolites, during fungal treatment. Further research is essential to thoroughly assess these effects. Therefore, *T. versicolor* can reduce 5 % HTL-AP COD, but not continuously; over time, there was an increase in COD that may be associated with products generated by the fungus.

Submerged cultivation of filamentous fungi has been extensively studied in the literature and integrated into industrial practices [52]. Various types of bioreactors have been investigated for filamentous fungi cultivation, such as stirred tanks, bubble-column, packed, and fluidized-bed reactors [52]. For instance, promising results have been achieved when using fluidized bed bioreactors to degrade pharmaceuticals by *T. versicolor* for wastewater treatment [53,54]. In this study, three days of *T. versicolor* HTL-AP treatment showed promising results in enhancing NO_3_^−^-N and NH_3_/NH_4_^+^-N concentrations in HTL-AP for future use as fertilizer on a bench scale. Future research should evaluate *T. versicolor* HTL-AP bioremediation using different bioreactor configurations to determine optimal performance conditions for the fungus and assess the feasibility of this treatment when scaling up. Additionally, HTL-AP composition can change depending on the HTL feedstock [55], which may alter fungal performance during treatment. Evaluating the impact of different HTL-AP compositions on fungal treatment will also be crucial to assessing the feasibility of employing *T. versicolor* in this context. Thus, such investigations are essential for the successful implementation of this biotechnological approach.

### 3.2 Wastewater nitrifying experiment

**Fig. 4:**
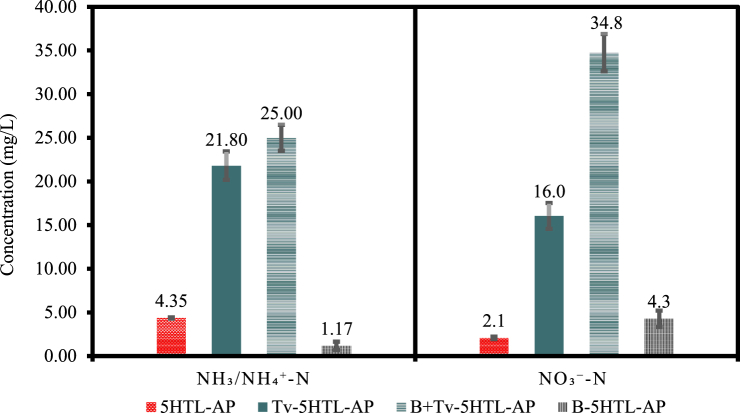
Comparison of NO_3_^−^-N and NH_3_/NH_4_^+^-N concentrations between the HTL-AP negative control (5HTL-AP) and HTL-AP treated with *Trametes versicolor* (Tv-5HTL-AP), *Trametes versicolor* and nitrifying bacteria (B + Tv-5HTL-AP), and nitrifying bacteria alone (B-5HTL-AP).

The addition of nitrifying bacteria to HTL-AP after fungal treatment may be beneficial to assist with NH_4_^+^ conversion into NO_3_^−^, as previously discussed. During nitrification, ammonia-oxidizing bacteria convert NH_3_ to NO_2_^−^, which is further converted to NO_3_^−^ by nitrate-oxidizing bacteria [56]. An attempt to apply nitrifying bacteria to increase NO_3_^−^ concentration in 5 % HTL-AP from swine manure has been reported; however, only a 1.75 mg/L increase in NO_3_^−^ + NO_2_^−^ concentration was observed [7]. In contrast, we added nitrifying bacteria to the HTL-AP after fungal treatment and compared the outcomes with those obtained from HTL-AP treated with the isolated microbes (Fig. 4).

HTL-AP samples inoculated with *Trametes versicolor* resulted in a noticeable NH_3_/NH_4_^+^-N concentration increase (Fig. 4), which aligns with results from section 3.1. Bacteria and fungus (B + Tv-5HTL-AP) and fungus (Tv-5HTL-AP) treatments presented values of NO_3_^−^-N and NH_3_/NH_4_^+^-N significantly higher than bacteria treatment (B-5HTL-AP) and no treatment (5HTL-AP) (Table 3). An approximately five-times increase in NH_3_/NH_4_^+^-N was found for samples containing the fungus, with (B + Tv-5HTL-AP) or without (Tv-5HTL-AP) bacteria addition, compared to the control (5HTL-AP). Once again, it is hypothesized that the fungus produced NH_3_/NH_4_^+^ through ammonification. In contrast, the sample only inoculated with bacteria (B-5HTL-AP) decreased the NH_3_/NH_4_^+^-N concentration 3.7-times compared to the control, which may indicate the microbes have performed nitrification. This claim agrees with the increase in NO_3_^−^-N for the sample containing only bacteria in 5 % HTL-AP (B-HTL-AP). The concentration of NO_3_^−^-N increased in all treated samples. The results from samples containing bacteria, fungus and the combination of both resulted in NO_3_^−^-N concentration increase of 2, 8 and 17 times, respectively, compared to the control. Compared to the fungal treatment (Tv-5HTL-AP), a two-times increase was observed when nitrifying bacteria were added to the treatment (B + Tv-5HTL-AP). These results suggest that the addition of bacteria has a beneficial impact on the treatment for the specific objectives of this study. Thus, it is likely that the fungus is performing ammonification, releasing NH_3_/NH_4_^+^ during the treatment, while bacteria are taking up this inorganic nitrogen and converting it to NO_3_^−^.

**Table 3:** Tukey HSD test for NO_3_^−^-N and NH_3_/NH_4_^+^-N as a function of treatment.

Treatment	NO_3_^−^-N		NH_3_/NH_4_^+^-N	
	mg/L	Tukey HSD	mg/L	Tukey HSD
B + Tv-5HTL-AP	34.8 ± 2.1	a	25 ± 1.5	a
Tv-5HTL-AP	16 ± 1.5	b	21.8 ± 1.6	a
B-5HTL-AP	4.3 ± 0.9	c	1.2 ± 0.5	b
5HTL-AP	2.1 ± 0.1	c	4.4 ± 0.1	b

Note: within each column, means that are followed by the same letter are not significantly different (*p* < 0.05).

Legend: B + Tv-5HTL-AP – fungal treatment with nitrifying bacteria addition, Tv-5HTL-AP – fungal treatment, B-5HTL-AP – HTL-AP treated with only nitrifying bacteria, and 5HTL-AP - negative control.

## 4 Conclusion

HTL-AP valorization will benefit the overall value and efficiency HTL process. The use of this wastewater stream as a fertilizer is possible, but requires increasing inorganic nitrogen availability. Here, we demonstrated that a 5 % HTL-AP solution treated with the white-rot fungus *T. versicolor* for 3 days increased NO_3_^−^-N concentration from 1.84 mg/L to 30.67 mg/L, and NH_3_/NH_4_^+^-N from 4.04 mg/L to 32.20 mg/L, representing an increase of almost 17- and 8-times, respectively. It is also worth noting that the introduction of nitrifying bacteria into the 3-day fungal treatment resulted in a two-fold increase of NO_3_^−^-N compared to fungal treatment alone. Thus, *T. versicolor* is a great candidate to increase inorganic nitrogen in 5 % HTL-AP; nonetheless, more research is needed to assess the wastewater toxicity and growth of hydroponic plants after the fungal treatment.

## Data availability statement

No data associated with this study has been deposited into a publicly available repository. Data will be made available on request.

## CRediT authorship contribution statement

**Vitoria F. C. Leme:** Writing – original draft, Visualization, Validation, Methodology, Investigation, Formal analysis, Data curation, Conceptualization. **Karla Lopez:** Investigation, Data curation. **Tiago Costa:** Formal analysis, Data curation. **Beth Conerty:** Writing – review & editing. **Laurie B. Leonelli:** Writing – review & editing. **Yuanhui Zhang:** Writing – review & editing. **Paul C. Davidson:** Writing – review & editing, Validation, Supervision, Resources, Project administration, Funding acquisition, Formal analysis, Data curation, Conceptualization.

## Declaration of competing interest

The authors declare the following financial interests/personal relationships which may be considered as potential competing interests: Paul C Davidson reports financial support was provided by 10.13039/100000199USDA 10.13039/100005825National Institute of Food and Agriculture. If there are other authors, they declare that they have no known competing financial interests or personal relationships that could have appeared to influence the work reported in this paper.
